# Supplementary material for: Tetrahedral triple-Q magnetic ordering and large spontaneous Hall conductivity in the metallic triangular antiferromagnet Co1/3TaS2
Source: Nat Commun. 2023 Dec 15;14:8346. doi: 10.1038/s41467-023-43853-4 (PMC10724158; doi:10.1038/s41467-023-43853-4)
Supplement: Supplementary file 1 — Supplementary Information [file 41467_2023_43853_MOESM1_ESM.pdf]

Supplementary Information: Tetrahedral triple-Q magnetic ordering and large spontaneous Hall conductivity in the metallic triangular antiferromagnet $Co_{1/3}TaS_2$

Pyeongjae Park[1,2], Woonghee Cho[1,2], Chaebin Kim[1,2], Yeochan An[1,2], Yoon-Gu Kang[3], Maxim Avdeev[4,5], Romain Sibille[6], Kazuki Iida[7], Ryoichi Kajimoto[8], Ki Hoon Lee[9], Woori Ju[10], En-Jin Cho[10], Han-Jin Noh[10], Myung Joon Han[3], Shang-Shun Zhang[11], Cristian D. Batista[12,13*], and Je-Geun Park[1,2,14*]

[1]*Center for Quantum Materials, Seoul National University; Seoul 08826, Republic of Korea*
[2]*Department of Physics & Astronomy, Seoul National University; Seoul 08826, Republic of Korea*
[3]*Department of Physics, KAIST; Daejeon 34141, Republic of Korea*
[4]*Australian Nuclear Science and Technology Organisation (ANSTO); New Illawarra Road, Lucas Heights, NSW 2234, Australia*
[5]*School of Chemistry, The University of Sydney; Sydney, NSW 2006, Australia*
[6]*Laboratory for Neutron Scattering and Imaging, Paul Scherrer Institut; 5232 Villigen, Switzerland*
[7]*Comprehensive Research Organization for Science and Society (CROSS); Tokai, Ibaraki 319-1106, Japan*
[8]*Materials and Life Science Division, J-PARC Center, Japan Atomic Energy Agency; Tokai, Ibaraki 319-1195, Japan*
[9]*Department of Physics, Incheon National University; Incheon, 22012, Republic of Korea*
[10]*Department of Physics, Chonnam National University; Gwangju 61186, Republic of Korea*
[11]*School of Physics and Astronomy and William I. Fine Theoretical Physics Institute, University of Minnesota, Minneapolis, MN 55455, USA*
[12]*Department of Physics and Astronomy, The University of Tennessee; Knoxville, Tennessee 37996, USA*
[13]*Quantum Condensed Matter Division and Shull-Wollan Center, Oak Ridge National Laboratory; Oak Ridge, Tennessee 37831, USA*
[14]*Institute of Applied Physics, Seoul National University; Seoul 08826, Republic of Korea*

* Corresponding author: cbatist2@utk.edu & jgpark10@snu.ac.kr

Supplementary Notes

Two different reports on the magnetic ordering wave vector of $Co_{1/3}TaS_2$: $\mathbf{q}_m$ = (1/2, 0, 0) and (1/3, 1/3, 0). As described in the main text, a previous neutron diffraction study[1] reported an ordering wave vector $\mathbf{q}_m = \pm(1/3, 1/3, 0)$ in $Co_{1/3}TaS_2$. However, as clearly shown in Fig. 2, the magnetic Bragg peaks of our $Co_{1/3}TaS_2$ samples appear at the M points ($\mathbf{q}_m$ = (1/2, 0, 0) and symmetry related vectors) of the Brillouin zone instead of the K points $\mathbf{q}_m = \pm(1/3, 1/3, 0)$. A natural explanation would be that the sample used in Ref.1 May exhibit substantial variations from the one utilized in our work with regard to sample quality and the presence of Co disorder". In this note, we offer a thorough analysis, leading to the conclusion that our observation of $\mathbf{q}_m$ = (1/2, 0, 0) and the occurrence of spontaneous Hall conductivity can be attributed to the nearly ideal stoichiometry of $Co_{1/3}TaS_2$, suggesting that these phenomena are intrinsic to the material.

It is reasonable to consider that varying cobalt compositions could be the primary source of the divergent results. Through an exhaustive investigation of composition dependence, we confirmed that the key bulk properties presented in our manuscript – ordering wave vector $\mathbf{q}_m$ = (1/2, 0, 0), two successive phase transitions, large spontaneous Hall conductivity, and weak ferromagnetic moment – remain intact for Co_xTaS_2 with $0.299(4) < x < 0.325(4)$. In other words, they can be found in the sample with Co compositions around the stoichiometric limit $x = 1/3$, but also remain qualitatively the same across a wide Co vacancy concentration of 2.5% ~ 10 %. This indicates that the observed properties are intrinsic phenomena rather than a consequence of Co vacancy effects. Some of these results are already demonstrated in our previous study[2] which reported a large spontaneous Hall conductivity in $Co_{1/3}TaS_2$ for the first time (see its Supplementary Materials of that work). Also, as seen in Fig. 2f of Ref. [2], $Co_{1/3}TaS_2$ with slightly different Co compositions show nearly the same magnitude of anomalous Hall conductivity ($\sigma_{xy}(\mathbf{H} = 0)$) independently of the longitudinal conductivity (σ_{xx}), indicating its intrinsic origin and small correlation with Co composition.

Meanwhile, the estimated Co composition of Ref. [1] 's sample can be found in Ref. [3]: $x \sim 0.29$. While the accuracy of this value remains uncertain, it is important to note that it falls outside the range we explored ($0.299(4) < x < 0.325(4)$). Furthermore, this composition deviates significantly from the ideal stoichiometry of $x = 1/3$. Therefore, it may be necessary to explore a broader range of cobalt compositions, particularly those more distant from $x = 1/3$, in order to detect $\mathbf{q}_m$ = (1/3, 1/3, 0).

Except for the composition $x = 0.29$, there is no further published information about the structural characterization of Ref. [1] 's sample, such as X-ray or neutron (nuclear) diffraction profile. Hence, we were unable to conduct a more extensive comparison beyond composition. Instead, we provide a detailed account of our assessment protocol for Co disorder in our samples, including the selection of the least disordered sample. This additional information complements the methodology already outlined in the Methods section.

Given the inherent uncertainties associated with vacancy analysis and the possibility of disorder in the XRD/Raman profiles, we have determined that evaluating sample quality through magnetometry measurements of the transition temperature (T_{N2}) offers greater sensitivity and accuracy. This observation stems from our discovery that T_{N2} progressively decreases with a reduction in x, and it would exhibit variations when factors such as Co disorder, aside from vacancies, are introduced. Unless the departure from ideal stoichiometry is substantial, T_{N2} has been consistently measured at

26.5 K, marking the highest value we have encountered across approximately 40 batches of single-crystal $Co_{1/3}TaS_2$ synthesis in our extensive experience. All measurements detailed in this manuscript were exclusively conducted using powder and single-crystal $Co_{1/3}TaS_2$ samples, all of which exhibited a T_{N2} of 26.5 K. It is worth noting, however, that samples with slightly lower T_{N2} values display qualitatively similar bulk properties.

Magnetic orderings compatible with neutron diffraction and transport data on $Co_{1/3}TaS_2$. In this section, we explain how the combined transport (non-zero $\sigma_{xy}(\mathbf{H} = 0)$) and neutron diffraction data lead us to conclude that the low-temperature phase below T_{N2} = 26.5 K is a non-coplanar triple-**Q** ordering, or "tetrahedral ordering" in real space (note that the tetrahedron spanned by the spins of four magnetic sublattices does not need to be equilateral; see the next Note). These arguments only hold when the magnetic ordering wave vectors correspond to the M points (half of reciprocal lattice vectors) of the first Brillouin zone: $\mathbf{q}_{\mathrm{m}}^{1}$ = (1/2, 0, 0), $\mathbf{q}_{\mathrm{m}}^{2}$ = (-1/2, 1/2, 0), and $\mathbf{q}_{\mathrm{m}}^{3}$ = (0, -1/2, 0) in reciprocal lattice units.

As already described in the main text, a single-**Q** ordering contradicts the measured non-zero $\sigma_{xy}(\mathbf{H} = 0)$ and non-zero $M_z(\mathbf{H} = 0)$ due to its invariance under time reversal symmetry combined with lattice translation ($\equiv \tau_{1a}T$, see Fig. 2g or 2h). A double-**Q** ordering generally does not possess this symmetry. However, it still possesses other residual symmetries that preclude a finite $\sigma_{xy}(\mathbf{H} = 0)$[4]. Therefore, triple-**Q** ordering is the only scenario compatible with non-zero $\sigma_{xy}(\mathbf{H} = 0)$ and non-zero $M_z(\mathbf{H} = 0)$. The triple-**Q** ordering can be further divided into three different cases: collinear, co-planar (but non-collinear), and non-coplanar. Each possibility can be distinguished by analyzing the neutron diffraction data.

The most general magnetic ordering that has diffraction peaks at the M-points $\mathbf{q}_{\mathrm{m}}^{1}$ = (1/2, 0, 0), $\mathbf{q}_{\mathrm{m}}^{2}$ = (-1/2, 1/2, 0), and $\mathbf{q}_{\mathrm{m}}^{3}$ = (0, -1/2, 0) is

$$\mathbf{M}(\mathbf{r}_i) = \sum_{\nu=1}^{3} \mathbf{\Delta}_\nu \cos(\mathbf{q}_{\mathrm{m}}^{\nu} \cdot \mathbf{r}_i). \qquad (1)$$

where i is a site index. The vector Fourier components $\mathbf{\Delta}_\nu$ can be restricted by the refinement of neutron diffraction data. When using the group representation analysis based on the $P6_322$ space group and $\mathbf{q}_{\mathrm{m}}^{\nu}$, $\mathbf{\Delta}_\nu$ directly corresponds to the basis vectors of possible irreducible representations. Hereafter, $\mathbf{V}_{jk}^{\nu}$ denotes the kth basis vector of the Γ_j irreducible representation with respect to $\mathbf{q}_{\mathrm{m}}^{\nu}$. Note that $\mathbf{V}_{ij}^{\nu}$ with the same i and j but different ν (= 1, 2, 3) are related by the three-fold rotation with respect to the c-axis (C_{3z}). Supplementary Table 3 shows the basis vectors $\mathbf{V}_{jk}^{1}$ as an example, and some of them with $\mathbf{\Delta}_2 = \mathbf{\Delta}_3 = 0$ (*i.e.,* single-**Q**) are illustrated in Supplementary Figs. 7a–c.

For $Co_{1/3}TaS_2$, the basis vectors different from $\mathbf{V}_{22}^{\nu}$ (Γ_2 irreducible representation) and $\mathbf{V}_{41}^{\nu}$ (Γ_4 irreducible representation) in Supplementary Table 3 can be excluded because they lead to a strong disagreement with our diffraction data in Fig. 2. For example, they yield a high intensity at [0.5, 0.5, 1] (r.l.u.) or its symmetry-equivalent positions, while our data shows zero intensity at [0.5, 0.5, 1] and its symmetry-equivalent positions within the measurement error (Fig. 2d). The cases $\mathbf{\Delta}_1 = \mathbf{V}_{22}^{1}$ and

$\mathbf{\Delta}_1 = \mathbf{V}_{41}^1$ are illustrated in Supplementary Figs. 7a and 7c, respectively. Among these two remaining options, only the $\mathbf{V}_{22}^{\nu}$ ($\mathbf{V}_{41}^{\nu}$) component can generate finite intensity at (0, 0.5, 0) ([0.5, 0, 3]) or its symmetry-equivalent M points. In our data, both peaks are present at $T < T_{N2}$ (see Fig. 2d and Supplementary Fig. 6). Therefore, the magnetic structure of $Co_{1/3}TaS_2$ for $T < T_{N2}$ should be expressed as a linear combination of $\mathbf{V}_{22}^{\nu}$ and $\mathbf{V}_{41}^{\nu}$, (e.g., $\mathbf{\Delta}_1 = \alpha\mathbf{V}_{22}^1 + \beta\mathbf{V}_{41}^1$), which is illustrated in Supplementary Fig. 7b. Indeed, this Fourier component (with $\beta/\alpha = \sqrt{2}$) gives a nice agreement with our powder neutron diffraction profile covering all observable magnetic reflections (Fig. 2a). Meanwhile, the magnetic structure for $T_{N2} < T < T_{N1}$ only consists of $\mathbf{V}_{22}^{\nu}$ since only (0, 0.5, 0) has a finite intensity in that temperature range.

As mentioned above, single-**Q** and double-**Q** orderings are incompatible with the finite spontaneous Hall conductivity found at $T < T_{N2}$. A triple-**Q** ordering that yields the same powder neutron diffraction profile as that of the above-mentioned single-**Q** ordering ($\mathbf{\Delta}_1 = \alpha\mathbf{V}_{22}^1 + \beta\mathbf{V}_{41}^1$) is:

$$\mathbf{M}(\mathbf{r}_i) = \sum_{\nu=1}^{3}(\alpha\mathbf{V}_{22}^{\nu} + \beta\mathbf{V}_{41}^{\nu})\cos(\mathbf{q}_{\mathrm{m}}^{\nu}\cdot\mathbf{r}_i). \quad (2)$$

This is because the same powder neutron diffraction profile can be explained by a superposition of three single-**Q** orderings with basis vectors of the same class but from different $\mathbf{q}_{\mathrm{m}}^{\nu}$, i.e., $\mathbf{M}^1(\mathbf{r}_i) = \mathbf{V}_{jk}^1\cos(\mathbf{q}_{\mathrm{m}}^1\cdot\mathbf{r}_i)$, $\mathbf{M}^2(\mathbf{r}_i) = \mathbf{V}_{jk}^2\cos(\mathbf{q}_{\mathrm{m}}^2\cdot\mathbf{r}_i)$, and $\mathbf{M}^3(\mathbf{r}_i) = \mathbf{V}_{jk}^3\cos(\mathbf{q}_{\mathrm{m}}^3\cdot\mathbf{r}_i)$, where j and k are the same for all three orderings. Figs. 7d–f show the triple-**Q** counterparts of Figs. 7a–c giving the same powder diffraction pattern, where each case consists of $\mathbf{\Delta}_\nu = \mathbf{V}_{22}^{\nu}$, $\mathbf{\Delta}_\nu = \alpha\mathbf{V}_{22}^{\nu} + \beta\mathbf{V}_{41}^{\nu}$ ($\beta/\alpha = \sqrt{2}$), and $\mathbf{\Delta}_\nu = \mathbf{V}_{41}^{\nu}$. This demonstrates that $\mathbf{V}_{22}^{\nu}$ and $\mathbf{V}_{41}^{\nu}$ generate an out-of-plane collinear and in-plane co-planar triple-**Q** ordering, respectively. The case of $\mathbf{\Delta}_\nu = \alpha\mathbf{V}_{22}^{\nu} + \beta\mathbf{V}_{41}^{\nu}$ ($\beta/\alpha = \sqrt{2}$), which is the case of $Co_{1/3}TaS_2$ according to its neutron diffraction data, is non-coplanar (see Supplementary Fig. 7e). Note that the three vectors $\mathbf{\Delta}_\nu = \alpha\mathbf{V}_{22}^{\nu} + \beta\mathbf{V}_{41}^{\nu}$ ($\beta/\alpha = \sqrt{2}$) with $\nu = 1$, 2, 3 are orthogonal to each other (that is, $\mathbf{\Delta}_\nu \perp \mathbf{\Delta}_{\nu'}$ for $\nu \neq \nu'$), while the sets $\mathbf{\Delta}_\nu = \mathbf{V}_{41}^{\nu}$ and $\mathbf{\Delta}_\nu = \mathbf{V}_{22}^{\nu}$ are not; see Supplementary Figs 7g–i. Therefore, the non-coplanar triple-**Q** structure is the only magnetic ordering compatible with our neutron diffraction data and with non-zero $\sigma_{xy}(\mathbf{H} = 0)$ at $T < T_{N2}$. We note that a partially-ordered triple-**Q** structure, where one of the four magnetic sublattices is disordered, can be excluded because it is co-planar (e.g., see Supplementary Fig. 7f); if it is non-coplanar, the net magnetization is always non-zero, and therefore, the structure is no longer antiferromagnetic.

Equilateral and non-equilateral tetrahedral triple-Q orderings. After concluding that only a non-coplanar triple-**Q** ordering is compatible with our experimental data at $T < T_{N2}$, we discuss two types of non-coplanar triple-**Q** structures that can be compatible with our experimental data: equilateral and non-equilateral tetrahedral orderings.

The magnetic moment of the equilateral tetrahedral triple-**Q** state – the most symmetric non-coplanar triple-**Q** structure – corresponds to $\mathbf{\Delta}_\nu \perp \mathbf{\Delta}_{\nu'}$ for $\nu \neq \nu'$ and $|\mathbf{\Delta}_\nu| = |\mathbf{\Delta}_{\nu'}|$:

$$\mathbf{M}_i^{\mathrm{tetr}} = \sum_{\nu=1}^{3} \boldsymbol{\Delta}_\nu \cos(\mathbf{q}_{\mathrm{m}}^{\nu} \cdot \mathbf{r}_i). \tag{3}$$

The triple-**Q** state described by $\mathbf{M}_i^{\mathrm{tetr}}$ yields precisely the same neutron diffraction pattern as that from the combination of three single-**Q** states $\mathbf{M}_\nu(\mathbf{r}_i)$ ($\nu = 1, 2, 3$). The conditions $\boldsymbol{\Delta}_\nu \perp \boldsymbol{\Delta}_{\nu'}$ for $\nu \neq \nu'$ and $|\boldsymbol{\Delta}_\nu| = |\boldsymbol{\Delta}_{\nu'}|$ guarantee that $\mathbf{M}_i^{\mathrm{tetr}}$ consists of a four sublattice structure, whose magnetic moments point along principal directions of a regular tetrahedron (Fig. 1a). While the tetrahedron can be freely rotated for the ground state of an isotropic model, it is helpful to consider the simple example $\boldsymbol{\Delta}_1 = \hat{x}$, $\boldsymbol{\Delta}_2 = \hat{y}$, and $\boldsymbol{\Delta}_3 = \hat{z}$ (see Fig. 1a in Ref. [5]). In the case of $Co_{1/3}TaS_2$, the Rietveld refinement gives $\boldsymbol{\Delta}_1 = \alpha\mathbf{V}_{22}^1 + \beta\mathbf{V}_{41}^1$), , and $\boldsymbol{\Delta}_{2,3}$ is subsequently derived based on the same basis vectors: $\boldsymbol{\Delta}_\nu = \alpha\mathbf{V}_{22}^\nu + \beta\mathbf{V}_{41}^\nu$. Or, equivalently, $\boldsymbol{\Delta}_2$ ($\boldsymbol{\Delta}_3$) can be obtained by rotating $\boldsymbol{\Delta}_1$ by 120° (240°) about the *c*-axis. In this case, the orthogonality condition is met only with the unique value of $\beta/\alpha = \sqrt{2}$, as demonstrated in Supplementary Fig. 8.

However, the magnitude of three Fourier components does not have to be the same in general ($|\boldsymbol{\Delta}_\nu| \neq |\boldsymbol{\Delta}_{\nu'}|$). If not equal, the tetrahedron characterizing the four principal directions is no longer equilateral (i.e. a non-equilateral *tetrahedral* triple-**Q** ordering). In the case of $Co_{1/3}TaS_2$ (i.e. $\beta/\alpha = \sqrt{2}$), this distortion breaks the C_{3z} rotation symmetry in the equilateral case. In principle, this possibility can be examined by comparing the intensities of the three magnetic Bragg peaks at $\mathbf{q}_{\mathrm{m}}^1$ = (1/2, 0, *L*), $\mathbf{q}_{\mathrm{m}}^2$ = (-1/2, 1/2, *L*), and $\mathbf{q}_{\mathrm{m}}^3$ = (0, -1/2, *L*) in a single-crystal neutron diffraction measurement. Since they are also connected by the C_{3z} symmetry, the equal (unequal) intensity of these three peaks directly corresponds to the equal (unequal) $\boldsymbol{\Delta}_\nu$.

Our experimental results (Fig. 2c and 4e) indicate that the intensities of these three Bragg peaks are identical within the measurement error, even under a magnetic field. Also, our simple phenomenological spin model (see the next note below), which reproduces the qualitative features of the measured INS data, predicts the equilateral tetrahedral ground state. This leads us to consider the equilateral tetrahedral ordering as the primary candidate to explain the observed phenomenology (anomalous transport and neutron diffraction). Nevertheless, the non-equilateral tetrahedral ordering cannot be excluded since the intensity of the three peaks can be identical due to the formation of three equally populated magnetic domains. For example, a combination of $c|\boldsymbol{\Delta}_1| = |\boldsymbol{\Delta}_2| = |\boldsymbol{\Delta}_3|$, $|\boldsymbol{\Delta}_1| = c|\boldsymbol{\Delta}_2| = |\boldsymbol{\Delta}_3|$, and $|\boldsymbol{\Delta}_1| = |\boldsymbol{\Delta}_2| = c|\boldsymbol{\Delta}_3|$ (*c* is an arbitrary coefficient within $0 < c < 1$), can give the same intensity.

Minimal phenomenological model Hamiltonian of $Co_{1/3}TaS_2$ and its phase diagram. In this section, we describe a minimal phenomenological model Hamiltonian that can capture the characteristic magnetic properties of $Co_{1/3}TaS_2$. This model can be used to describe the long-wavelength limit (a low energy excitation spectrum) of the problem under consideration because the corresponding non-linear sigma model has enough free parameters to control the shape and velocity of the Goldstone modes, as well as the gap of the accidental quadratic gapless mode. The Hamiltonian includes competing Heisenberg interactions, such that the Fourier transform of their sum, $J(\mathbf{k})$, has global minima at the M-points. A small bi-quadratic interaction between nearest neighbor (NN) spins

on the same layer is also added to split the continuous degeneracy between different multi-**Q** orderings and stabilize the tetrahedral triple-**Q** state:

$$H = \sum_{i,j_m} J_m \mathbf{S}_i \cdot \mathbf{S}_{j_m} + \sum_{i,j_c} J_c \mathbf{S}_i \cdot \mathbf{S}_{j_c} + \sum_{i,j_1} K_{\mathrm{bq}} (\mathbf{S}_i \cdot \mathbf{S}_{j1})^2. \quad (4)$$

The exchange interactions J_m connect m^{th} intralayer NN sites. In our minimal model, we only consider two competing exchange interactions J_1 and J_2. The strength of inter-layer NN interaction J_c controls the spin velocity along the direction perpendicular to the layers. Finally, the strength of the biquadratic interaction controls the energy gap of the quadratic gapless mode of the pure Heisenberg Hamiltonian, which arises from the above-mentioned continuous degeneracy between different multi-**Q** orderings present in any pure classical Heisenberg model when $J(\mathbf{k})$ has global minima at the M-points: any classical state $\mathbf{M}_i = \sum_{\nu=1}^{3} \mathbf{\Delta}_\nu \cos(\mathbf{q}_{\mathrm{m}}^{\nu} \cdot \mathbf{r}_i)$ with $\mathbf{\Delta}_\nu \perp \mathbf{\Delta}_{\nu'}$ for $\nu \neq \nu'$ and $\sum_{\nu=1}^{3} |\mathbf{\Delta}_\nu|^2 = S^2$ is a ground state.

A simple energy minimization via simulated annealing (see Methods) shows that the phase diagram of this model includes three common AFM orderings of the TLAF: stripe, 120°, and tetrahedral ground states (see left panel of Supplementary Fig. 8b). In addition to antiferromagnetic (AFM) J_1, AFM J_2 leads to a ground state with the ordering wave vector $\mathbf{q}_{\mathrm{m}}$ = (1/2, 0, 0) or its symmetry-related vectors. This $\mathbf{q}_{\mathrm{m}}$ is what we observed in $Co_{1/3}TaS_2$, and the negative Curie-Weiss temperature (~ -100 K) of $Co_{1/3}TaS_2$ is consistent with AFM J_1 and AFM J_2 [2]. As we already mentioned, the stripe and tetrahedral orderings are degenerate for $K_{\mathrm{bq}} = 0$ (Supplementary Fig. 8c). However, our analysis based on simulated annealing always yields a stripe ground state for $K_{\mathrm{bq}} = 0$ due a thermally induced order-by-disorder mechanism that favors collinear orderings[6,7] (see Supplementary Fig. 8b). A positive K_{bq} penalizes the collinear stripe ordering and stabilizes the tetrahedral triple-**Q** ordering (Supplementary Fig. 8b-c).

In addition, as mentioned in the main text, $Co_{1/3}TaS_2$ has a sizable AFM interlayer coupling. A simple LSWT fitting of our INS data in Fig. 3d leads to $J_c S^2$ ~ 2.95 meV. The effect of adding J_c on the magnetic phase diagram is demonstrated in the right panel of Supplementary Fig. 8b. Interestingly, J_c destabilizes the 120° order and induces multi-**Q** phases for very small or very large J_2/J_1. However, the energy landscape between the stripe and tetrahedral orderings remains the same as in the two-dimensional model ($J_c = 0$). Therefore, a sizable J_c does not modify the competition between the stripe and tetrahedral orderings. To better understand this competition, we investigated a finite-temperature phase diagram using classical Monte Carlo (MC) simulations (see Materials and Methods). Supplementary Fig. 8d and Supplementary Fig. 8e show the staggered magnetization and scalar spin chirality obtained from our simulations, which are order parameters for the stripe and tetrahedral state, respectively. Interestingly, for small K_{bq} ($< 0.05\, J_1$), the spin system undergoes two phase transitions: from a paramagnet to the stripe ordering (orange arrows) and from the stripe to the tetrahedral ordering (red arrows). This transition can be attributed to stabilization of collinear ordering by thermal fluctuations. Therefore, the stripe order appears at high temperatures with enough entropy gain to overcome the energy cost of a small positive K_{bq}. We note that this result is consistent with our experimental observation in $Co_{1/3}TaS_2$.

We calculated its spin-wave spectra (see Materials and Methods) and compared them with our experimental INS data to further test our simple phenomenological model. The measured magnon spectra at 5 K (Figs. S10a and S10d) indicate a nearly isotropic velocity of the linear magnon mode along the in-plane momentum directions. Figs. S10(B) and S10(E) illustrate the calculated spin-wave spectra of the tetrahedral ordering with four different J_2/J_1: 0.05, 0.15, 0.25, and 0.35. Notably, a nearly isotropic magnon velocity similar to our data is found over a wide range of J_2/J_1.

On the other hand, the calculated spectra based on the stripe ordering show anisotropic in-plane dispersion of the linear mode (Supplementary Fig. 10c and 10f), which is especially apparent from the spectra along the K-M_1-B line. While this anisotropy could, in principle, be eliminated by fine-tuning multiple exchange parameters, it is expected to be present in the general case. To understand its origin, it is convenient to consider the long wavelength limit ($|\mathbf{q}| \ll 1$) of the pure 2D Heisenberg model. The dispersion of the two Goldstone modes (centered at the $\mathbf{\Gamma}$ and $\mathbf{Q}_\nu$ points) for the single-$\mathbf{Q}_\nu$ phase is given by:

$$E_{\mathrm{G}}^{\mathrm{stripe}}(\mathbf{q}) = \sqrt{v_1^2 q_1^2 + v_2^2 q_2^2}, \tag{5}$$

where $\mathbf{q} = q_1\,\mathbf{e}_1 + q_2\,\mathbf{e}_2$, with $\mathbf{e}_1 \parallel \mathbf{Q}_\nu$ and $\mathbf{e}_2 \perp \mathbf{Q}_\nu$, $J(\mathbf{Q}_\nu + \mathbf{q}) = J(\mathbf{Q}_\nu) + a\, q_1^2 + b\, q_2^2$, $v_1 = 2S\sqrt{a[J(\mathbf{0}) - J(\mathbf{Q}_\nu)]}$ and $v_2 = 2S\sqrt{b[J(\mathbf{0}) - J(\mathbf{Q}_\nu)]}$. It is then clear that $v_1 \neq v_2$ for the generic case (i.e., without fine tuning) and that the ratio v_1/v_2 can vary between 0 and infinity for the stripe order. The accidental quadratic gapless modes, centered at the remaining M-points $\boldsymbol{k} = \boldsymbol{Q}_{\nu'} + \delta\boldsymbol{k}$ with $\nu' \neq \nu$, are also anisotropic in general. For instance, for $\nu = 3$ we have:

$$E_{\mathrm{quad}}^{\mathrm{stripe}}(\delta\mathbf{k}) = 2S\sqrt{\left[J(\boldsymbol{Q}_1 + \delta\boldsymbol{k}) - J(\boldsymbol{Q}_3)\right]\left[J(\mathbf{Q}_2 + \delta\boldsymbol{k}) - J(\mathbf{Q}_3)\right]}$$

On the other hand, the three Goldstone modes of the tetrahedral ordering centered at the three M-points $\mathbf{Q}_\nu$ are also generically anisotropic:

$$E_{\mathrm{G}}^{\mathrm{tetr}}(\mathbf{q}) = \sqrt{v_1^2 q_1^2 + v_2^2 k_2^2}, \tag{6}$$

with

$$\begin{aligned} v_1 &= S\sqrt{2/3\big(J(\mathbf{0}) - J(\mathbf{Q}_\nu)\big)(3a + b)}, \\ v_2 &= S\sqrt{2/3\big(J(\mathbf{0}) - J(\mathbf{Q}_\nu)\big)(a + 3b)}. \end{aligned} \tag{7}$$

However, in this case, the ratio $v_x/v_y = \sqrt{(3a + b)/(a + 3b)}$ is bounded by $\sqrt{1/3} \leq v_x/v_y \leq \sqrt{3}$, implying the anisotropy cannot be bigger than $\approx 70\%$. The accidental gapless quadratic mode of the tetrahedral phase is centered at the Γ-point and its 2D dispersion is isotropic:

$$E_{\text{quad}}^{tetr}(\mathbf{k}) = \frac{S}{2}\sqrt{(a+3b)(3a+b)}\left(k_x^2+k_y^2\right), \tag{8}$$

for $|\mathbf{k}| \ll 1$.

Another apparent discrepancy between the data and the magnon spectra of the stripe order is the intensity of the accidental quadratic magnon mode relative to the Goldstone modes at the same energy. The disagreement between the data and the calculation for the minimal 3D model of Eq. (4) is highlighted as orange dashed boxes in Supplementary Fig. 10.

We finally discuss the limitations of our minimal phenomenological model. As the RKKY interaction is a primary source of magnetic interactions in $Co_{1/3}TaS_2$, longer-range interactions J_m with $m > 2$ can be significant. Also, an effective spin model derived from a Kondo Lattice model should include all the symmetry-allowed 4-spin interactions[8]. In addition, renormalization effects from higher-order $1/S$ corrections are not included in our fit, implying that the value of K_{bq} extracted from the spin wave spectra and thermodynamic phase diagram is lower than the actual value[9]. Based on the correction introduced in Ref. [9], the underestimation factor for $S = 3/2$ is $(1 - 1/S + 1/(4S^2)) = 4/9$, which leads to $K_{\text{bq}} \sim 0.045\ J_1$ after the correction.

Symmetry-allowed single-ion anisotropy terms and the gapped linear magnon mode. While the phenomenological model given by Eq. (4) provides a consistent picture for our measurements of $Co_{1/3}TaS_2$, it cannot explain the measured energy gap of the Goldstone modes because of the absence of spin anisotropy (Supplementary Fig. 11a). Anisotropic terms are also required to explain the stabilization of the refined spin configuration (Fig. 2i), in which one of the four spins (S_α in Supplementary Fig. 11c) is parallel to the c-axis and the in-plane components of the other three spins ($S_{\beta,\gamma,\delta}$ in Supplementary Fig. 11c) point along the a^* or its symmetrically equivalent directions.

Assuming that the Co^{2+} ion in $Co_{1/3}TaS_2$ has spin-3/2, the only symmetry-allowed single-ion anisotropy (SIA) term is $K(S_z)^2$. However, this term still leaves a residual $O(2)$ symmetry implying that only two of the three Goldstone modes will be gapped out. Moreover, the three Goldstone modes remain gapless at the linear spin-wave level. Consequently, anisotropic exchange interactions must also be added to account for the observed gap of the Goldstone modes, and this gap can only be captured by including higher-order corrections (beyond linear spin waves) in the $1/S$ expansion. This is because the classical limit of theory has accidental continuous symmetries even in the presence of symmetry-allowed anisotropic terms.

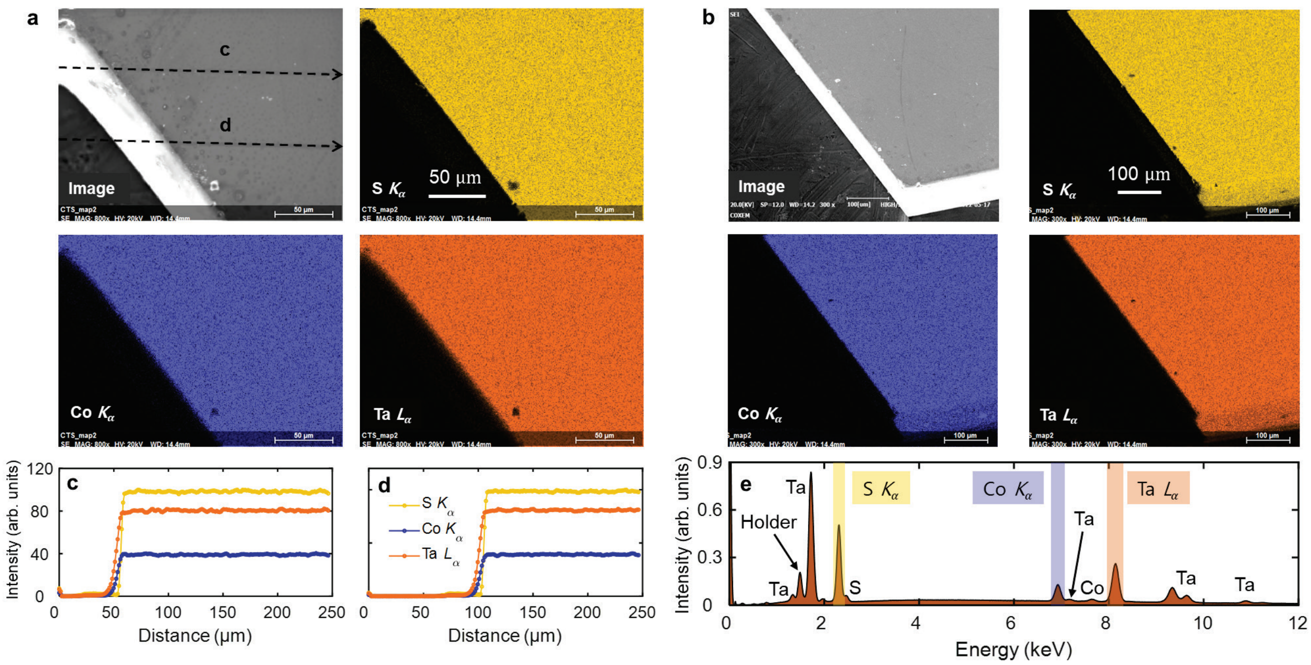

Supplementary Fig. 1 | Spatial distribution of Co, Ta, and S composition. a–b, Scanning electron microscope (SEM) image and EDX mapping of a single-crystal $Co_{1/3}TaS_2$ sample. **c–d,** Line profiles of the EDX mapping results in **a**, indicated as black dashed arrows in **a**. **e,** An EDX spectrum of $Co_{1/3}TaS_2$ with highlighted peaks of Co-K_α, Ta-L_α, and S-K_α edges tracked in the EDX mapping.

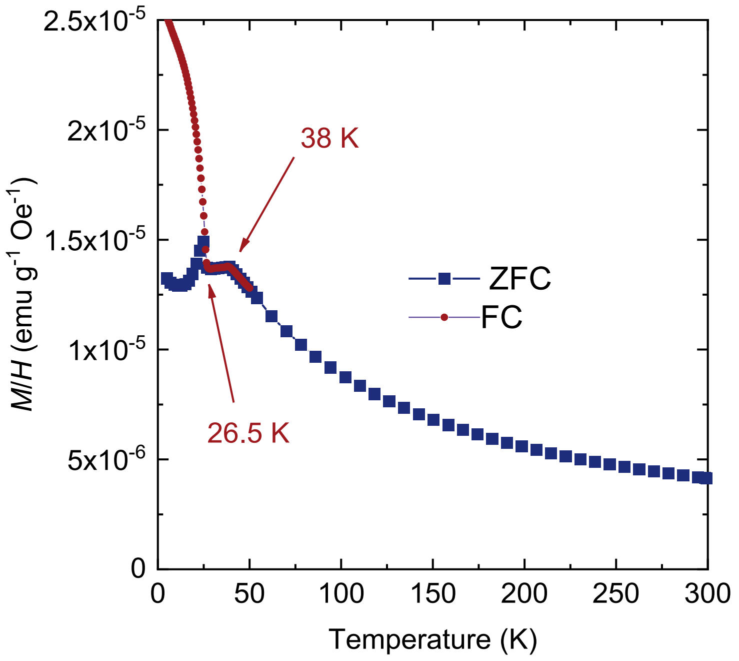

Supplementary Fig. 2 | Temperature-dependent magnetization of powder $Co_{1/3}TaS_2$ used in the powder neutron diffraction experiment.

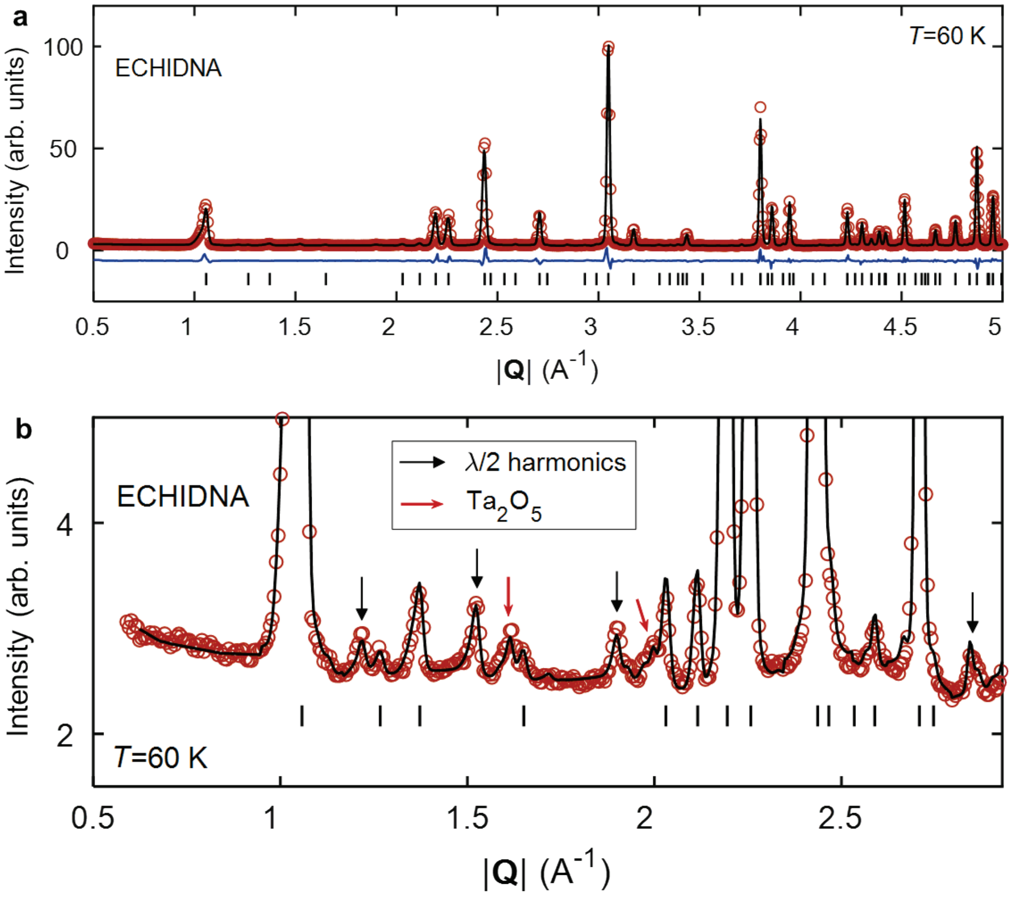

Supplementary Fig. 3 | **a,** Full powder neutron diffraction spectra of $Co_{1/3}TaS_2$ measured at 60 K. A solid blue line displays the difference between the data and the simulation. **b,** Low-angle part of **a**. Black solid lines show the simulation result from the Rietveld refinement (see Supplementary Table 2).

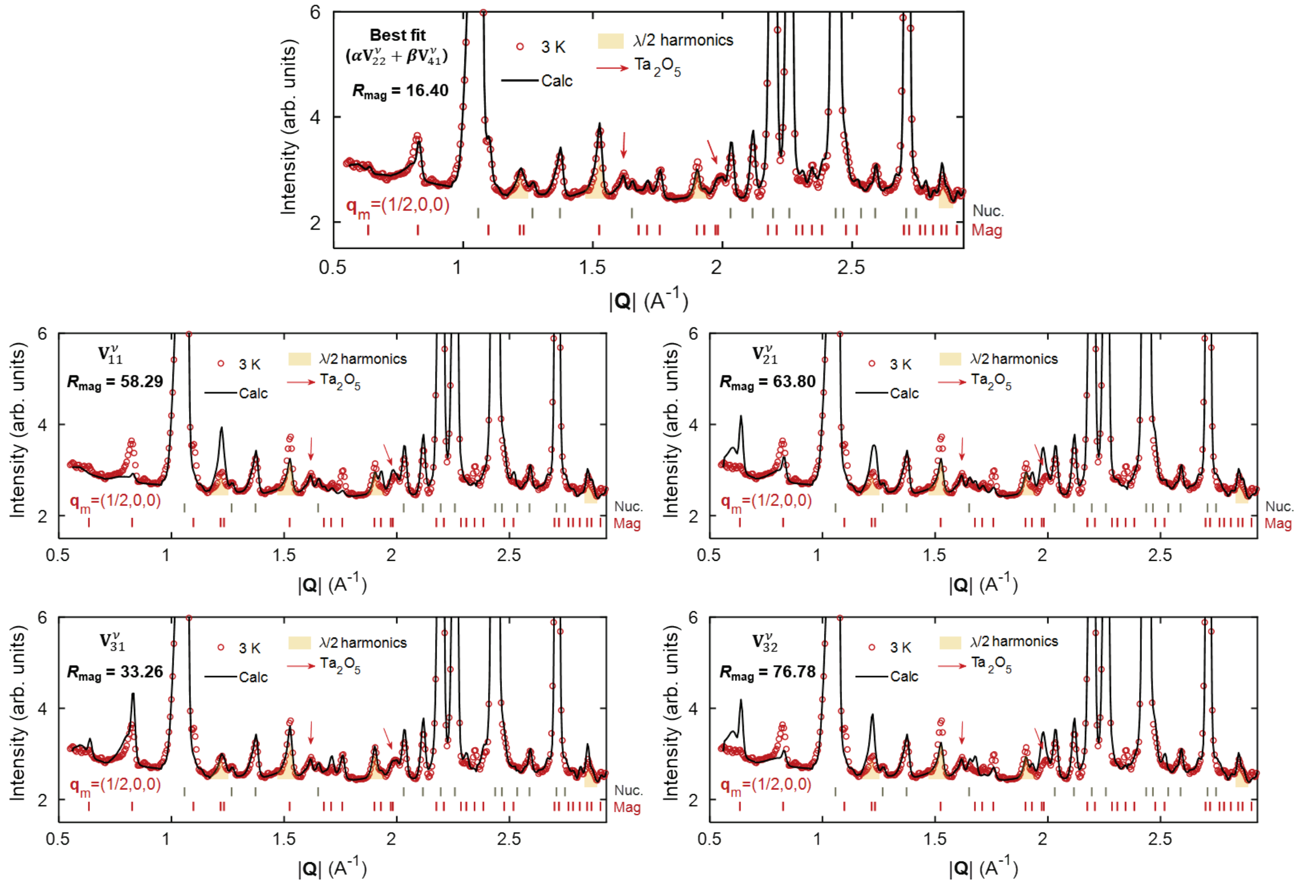

Supplementary Fig. 4 | Comparing the powder diffraction patterns of several different magnetic structure models. Grey (red) vertical solid lines denote the position of nuclear (magnetic) reflections. Black solid lines include both nuclear and magnetic structure factors. Basis vectors for each panel can be found in Supplementary Table 3.

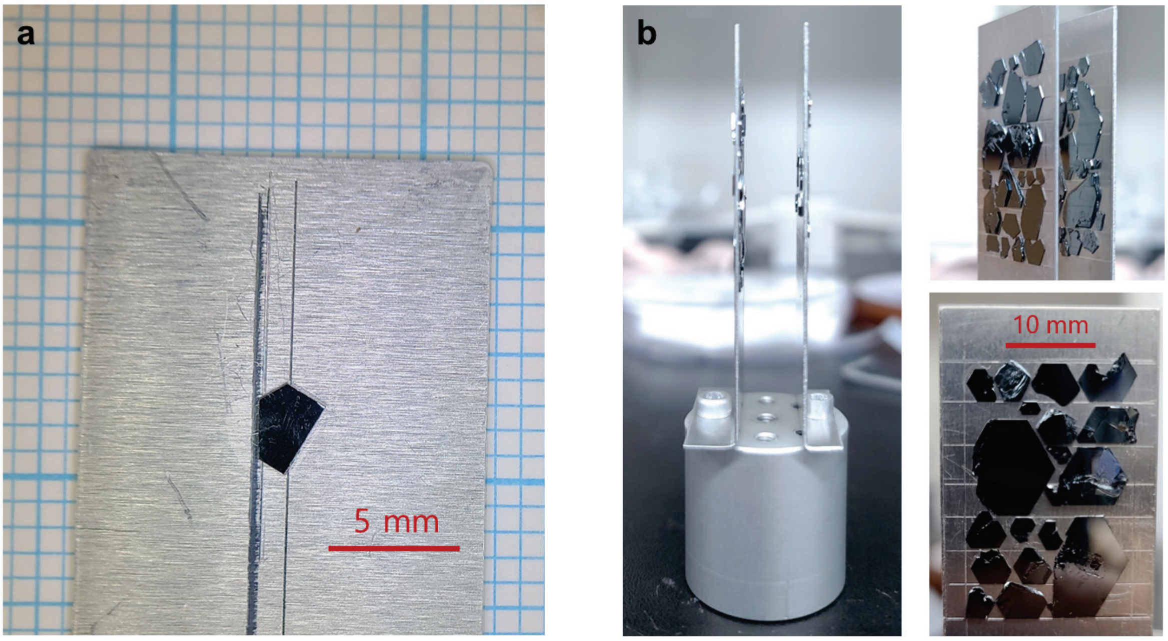

Supplementary Fig. 5 | a, Single-crystal $Co_{1/3}TaS_2$ for the neutron diffraction experiment at ZEBRA, PSI. **b,** Co-aligned single-crystal $Co_{1/3}TaS_2$ for the inelastic neutron scattering experiment at 4SEASONS, J-PARC. The overall mosaicity of the co-aligned crystals was ~ 1.5°.

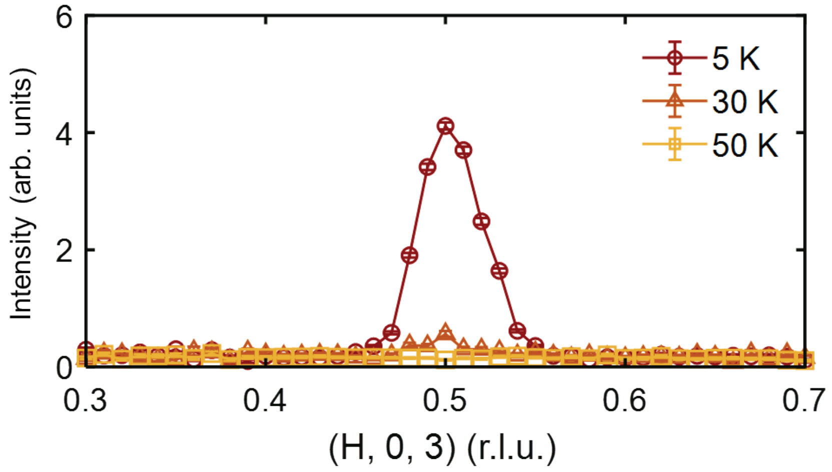

Supplementary Fig. 6 | Temperature dependence of the (0.5, 0, 3) magnetic Bragg peak, which corresponds to the presence of Γ_4 irreducible representation ($\mathbf{V}_{41}^{1}$, see Supplementary Table 2–3). Note that the (0.5, 0, 3) peak only exists below T_{N2} = 26.5 K. Error bars indicate the standard deviations of the data points.

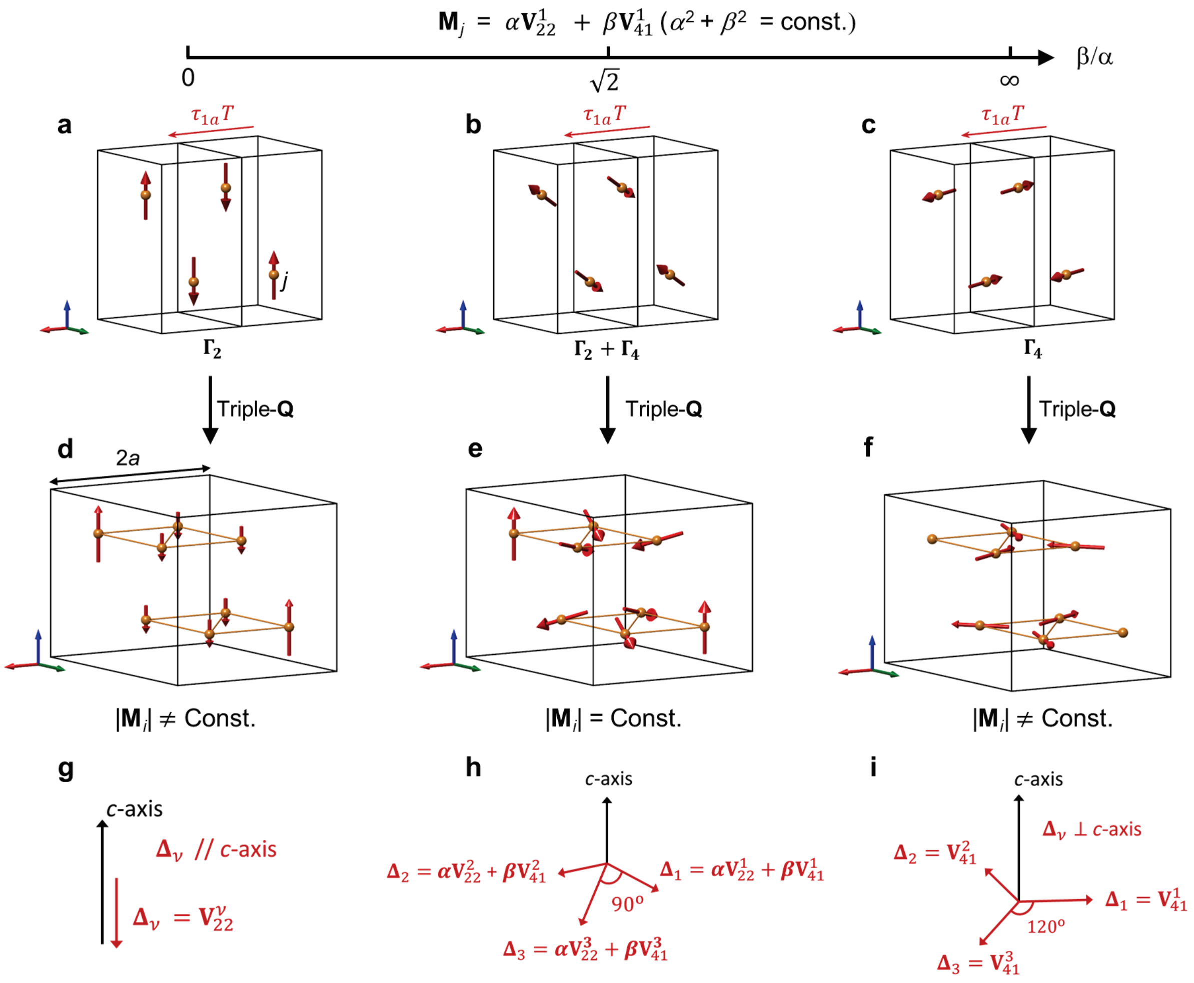

Supplementary Fig. 7 | a–c, Single-**Q** spin configurations of Γ_2, $\Gamma_2 + \Gamma_4$ ($\alpha\mathbf{V}_{22} + \beta\mathbf{V}_{41}$ with $\beta/\alpha = \sqrt{2}$, see Supplementary Table 4), and Γ_4 irreducible representations. **d–f,** Triple-**Q** counterparts of **a**–**c**. The equal magnitude of $\mathbf{M}_i$ for all i is satisfied only for a unique ratio between Γ_2 and Γ_4 (**b** and **e**). **g–i,** Three vector Fourier components $\boldsymbol{\Delta}_\nu$ that forms the triple-**Q** structure in **d**–**f** (see Supplementary Notes). Note that they are related to each other by the 120° rotation about the c-axis.

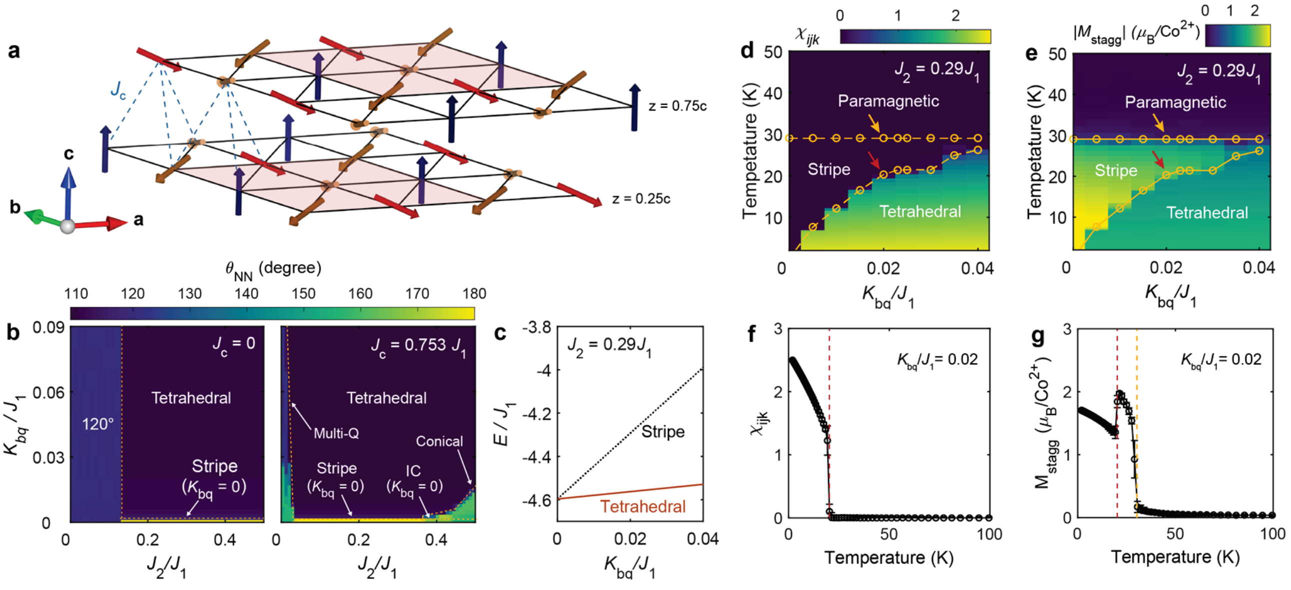

Supplementary Fig. 8 | Magnetic phase diagram of $Co_{1/3}TaS_2$ based on a minimal spin Hamiltonian. a, The interlayer spin configuration of $Co_{1/3}TaS_2$ with AB stacking, determined by antiferromagnetic NN inter-layer coupling (J_c > 0). The tetrahedral ordering in layer B (z = 0.75c) can be obtained by simply translating the magnetic ordering in layer A (z = 0.25c); see the red-shaded regions at z = 0.25c & 0.75c planes. **b**, Phase diagram of the (left) J_1-J_2-K_{bq} and (right) J_1-J_2-J_c-K_{bq} models calculated using simulated annealing (see Supplementary Notes). Color plots indicate the average intralayer NN spin angle of the calculated magnetic ground state. **c,** Comparison of the total energy between stripe (single-**Q**) and tetrahedral (triple-**Q**) ordering as a function of K_{bq}/J_1 with fixed J_2/J_1. **d–e** Finite-temperature phase diagram of the J_1-J_2-J_c-K_{bq} model with J_1S^2 = 3.92 meV, J_2/J_1 = 0.29, and J_c/J_1 = 0.753, calculated using classical Monte-Carlo (M-C) simulation. Color plots in **d** and **e** show the calculated $\chi_{ijk}(T)$ and $M_{\mathrm{stagg}}(T)$ from the MC simulation, respectively, which demonstrate two-step phase transitions for relatively small K_{bq}/J_1, consistent with our experimental observation. **f–g,** Calculated $\chi_{ijk}(T)$ and $M_{\mathrm{stagg}}(T)$ for K_{bq}/J_1 = 0.02 in **d–e**. Error bars in **f–g** indicate the standard deviations of the calculated quantities.

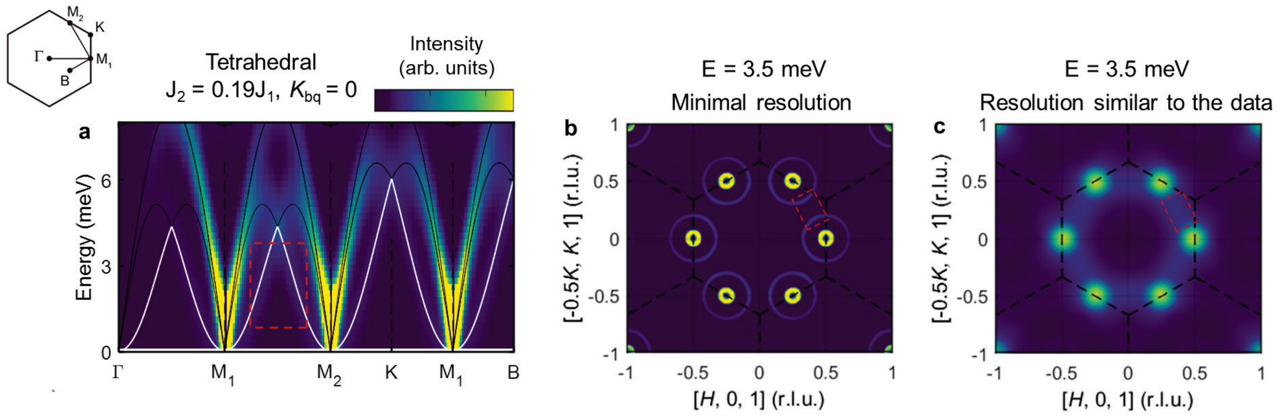

Supplementary Fig. 9 | Quadratic magnon mode. a, Calculated magnon dispersion and INS cross-section of the tetrahedral triple-**Q** ordering with J_1S^2 = 3.92 meV and J_cS^2 = 2.95 meV. Γ represents (001). The solid white line in **a** highlights the quadratic magnon mode. Note that the quadratic mode in **a** is gapless as we set K_{bq} = 0. **b–c,** Const-E cuts of the calculated magnon spectra in **a** without and with proper consideration of the resolution effect, respectively. The energy integration range is set to [3.3, 3.7] meV. With resolution convolution, the signal of the quadratic mode becomes blurred and takes on a simple line shape that connects the six M points.

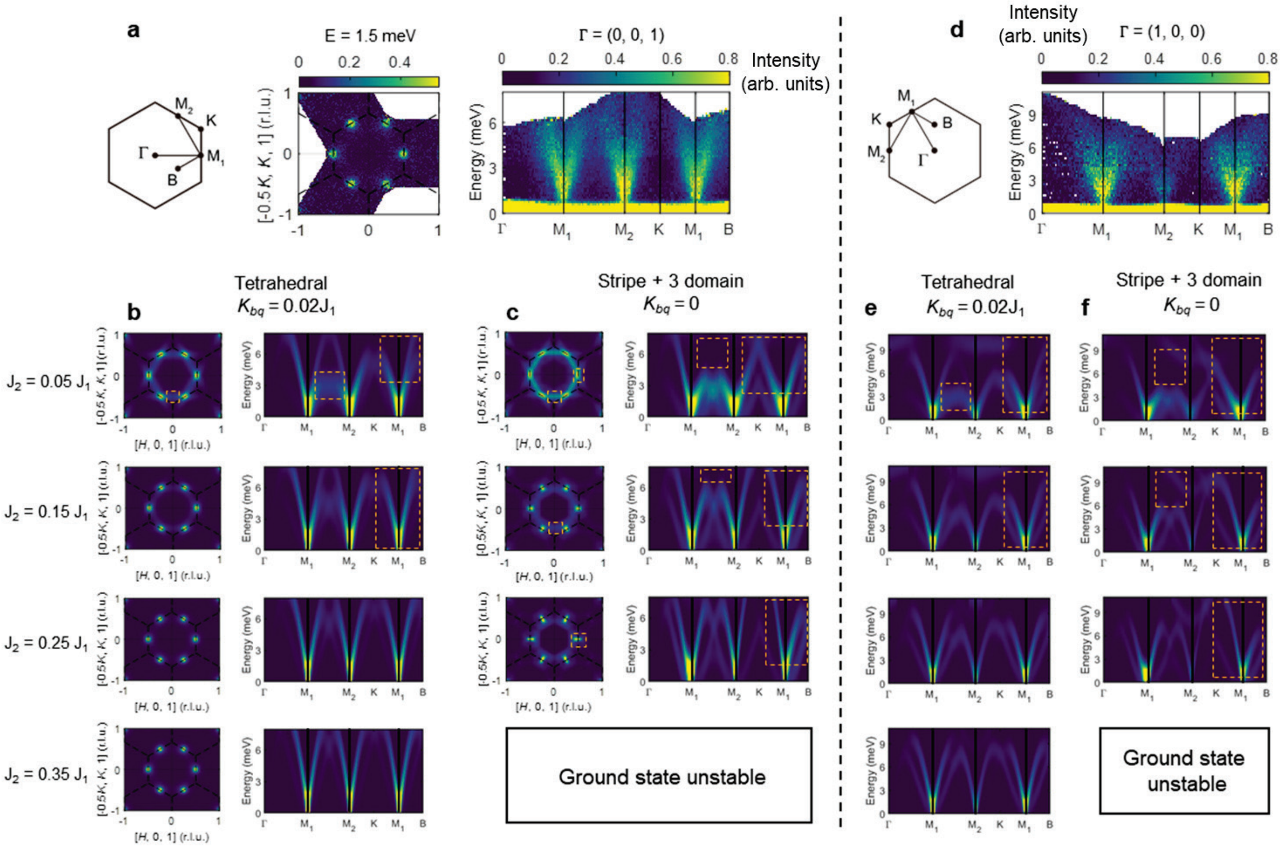

Supplementary Fig. 10 | A comprehensive comparison between measured and calculated magnon spectra of $Co_{1/3}TaS_2$. a, Constant-*E* cut and energy-momentum dispersion plot of the INS data measured at 5 K. Data with different incident neutron energies (E_i) are overlaid in the dispersion plot,. An energy integration range of the constant-*E* cut is set to [1.3, 1.5] meV. **b,** Calculated magnon spectra of the tetrahedral ordering based on the J_1-J_2-J_c-K_{bq} model, where J_1S^2 = 3.92 meV and J_cS^2 = 2.95 meV. Note that the signal of the linear mode is almost circular (*i.e.*, isotropic) for a wide range of J_2/J_1. **c,** Same as **b** but based on the stripe order with three magnetic domains. In this case, the linear mode's signal is highly anisotropic and has a circular shape (*i.e.*, isotropic) only for $J_2 \sim 0.1J_1$ (Fig. 3h). **d–f,** Same as **a–c**, but based on the magnon spectra in a different Brillouin zone $\mathbf{\Gamma} = (1, 0, 0)$. Orange dashed boxes in **b–f** indicate discrepancies between the data and the simulation, such as intensity, shape, and anisotropy of the linear mode's dispersion.

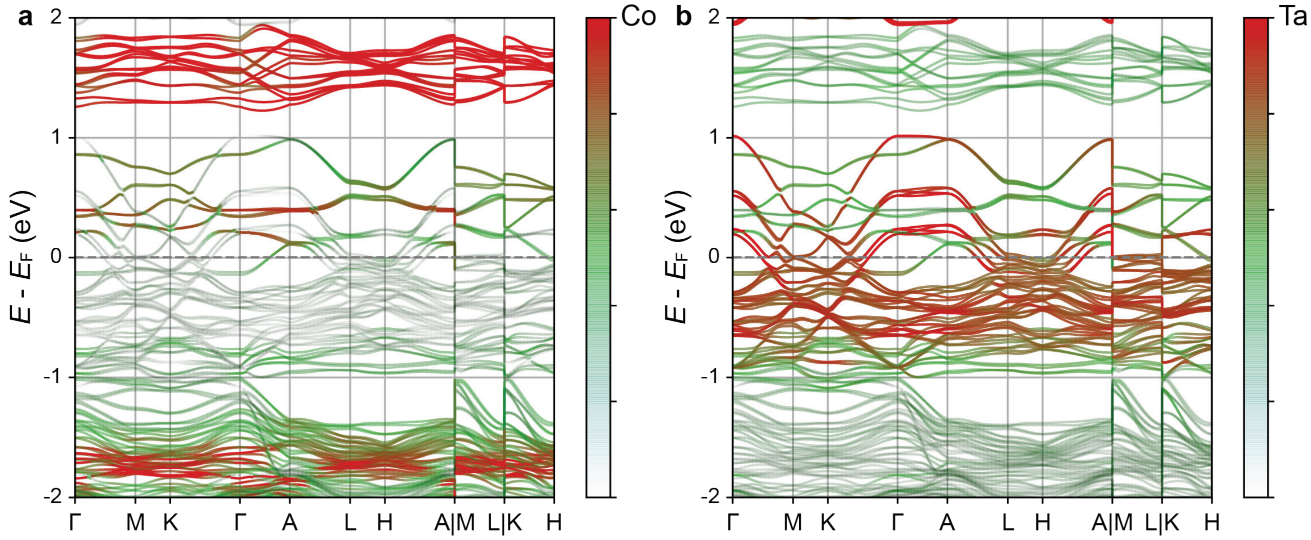

Supplementary Fig. 11 | a–b, Calculated electron band structure of $Co_{1/3}TaS_2$ with the tetrahedral triple-**Q** magnetic order in Fig. 4a. Color codes show the **a** Co-3*d* and **b** Ta-5*d* band portion.

Supplementary Table 1. The Rietveld refinement result of the powder neutron diffraction pattern for $T > T_N$ (Fig. 2a and Supplementary Fig. 3).

T = 60 K	Space group: $P\ 6_3\ 2\ 2$ (No. 182)<br>Cell dimensions: a=b=5.7255(1) Å, c=11.8783(1) Å, α=β=90°, γ=120°					
Atom	Wychoff letter	x/a	y/b	z/c	B_{iso} (Å^2)	Occupancy
Ta	2a	0	0	0	0.05	0.16667
Ta	4f	0.3333	0.6667	0.9991(3)	0.05	0.33333
Co	2c	0.3333	0.6667	0.25	0.10	0.1510
S	12i	0.6662(20)	0.0017(14)	0.1320(1)	0.10	0.9848(41)
	Agreement factors: R_p (%)=4.49, R_{wp} (%)=6.28, R_{exp} (%)=1.21, χ^2 =27.0					
	Ta_2O_5 : 0.76 %					

Supplementary Table 2. Character table of the $P6_322$ space group with the magnetic ordering wavevector of $\mathbf{q}_m^1$ = (1/2, 0, 0).

	1<br>(x, y, z)	$\{C_{2z} \mid \tau_{0.5c}\}$<br>(-$x$, -$y$, z+1/2)	C_{2a}<br>(-x, y-x, -z)	C_{2a^*}<br>(x, x-y, -z+1/2)
Γ_1	1	1	1	1
Γ_2	1	1	-1	-1
Γ_3	1	-1	1	-1
Γ_4	1	-1	-1	1

Supplementary Table 3. Basis vectors of the four irreducible representations (Irreps) in Supplementary Table 3. The basis vectors are formulated based on the lattice vectors **a**, **b,** and **c**.

Irreps	Basis vectors	Co1 (1/3, 2/3, 1/4)	Co2 (2/3, 1/3, 3/4)
Γ_1	$\mathbf{V}_{11}^{1}$	(2, 1, 0)	(2, 1, 0)
Γ_2	$\mathbf{V}_{21}^{1}$	(0, -1, 0)	(0, -1, 0)
	$\mathbf{V}_{22}^{1}$	(0, 0, 1)	(0, 0, -1)
Γ_3	$\mathbf{V}_{31}^{1}$	(0, -1, 0)	(0, 1, 0)
	$\mathbf{V}_{32}^{1}$	(0, 0, 1)	(0, 0, 1)
Γ_4	$\mathbf{V}_{41}^{1}$	(2, 1, 0)	(-2, -1, 0)

References

1 Parkin, S. S. P., Marseglia, E. A. & Brown, P. J. Magnetic structure of $Co_{1/3}NbS_2$ and $Co_{1/3}TaS_2$. *Journal of Physics C: Solid State Physics* **16**, 2765-2778 (1983).

2 Park, P. *et al.* Field-tunable toroidal moment and anomalous Hall effect in noncollinear antiferromagnetic Weyl semimetal $Co_{1/3}TaS_2$. *npj Quantum Materials* **7**, 42 (2022).

3 Parkin, S. S. P. & Friend, R. H. 3d transition-metal intercalates of the niobium and tantalum dichalcogenides. II. Transport properties. *Philosophical Magazine B* **41**, 95-112 (1980).

4 Kim, K.-S., Lee, K. H., Chung, S. B. & Park, J.-G. Magnon topology and thermal Hall effect in trimerized triangular lattice antiferromagnet. *Physical Review B* **100**, 064412 (2019).

5 Martin, I. & Batista, C. Itinerant electron-driven chiral magnetic ordering and spontaneous quantum Hall effect in triangular lattice models. *Physical Review Letters* **101**, 156402 (2008).

6 Henley, C. L. Ordering due to disorder in a frustrated vector antiferromagnet. *Physical Review Letters* **62**, 2056-2059 (1989).

7 Villain, J., Bidaux, R., Carton, J.-P. & Conte, R. Order as an effect of disorder. *Journal de Physique* **41**, 1263-1272 (1980).

8 Akagi, Y., Udagawa, M. & Motome, Y. Hidden Multiple-Spin Interactions as an Origin of Spin Scalar Chiral Order in Frustrated Kondo Lattice Models. *Physical Review Letters* **108**, 096401 (2012).

9 Dahlbom, D. *et al.* Renormalized Classical Theory of Quantum Magnets. *arXiv preprint arXiv:2304.03874* (2023).
